# Critical evaluation of the guidelines of the Finnish Advisory Board on Research Integrity and of their application

**DOI:** 10.1186/s41073-016-0020-9

**Published:** 2016-10-17

**Authors:** Liisa Räsänen, Erja Moore

**Affiliations:** Freelance researchers, Jyväskylä, Finland

**Keywords:** Research misconduct, Guidelines on research integrity, Finnish Advisory Board on Research Integrity, TENK

## Abstract

We have national guidelines for the responsible conduct of research (RCR) and procedures for handling allegations of misconduct in Finland. The guidelines have been formulated and updated by the Finnish Advisory Board on Research Integrity (TENK). In this article, we introduce and evaluate the national RCR guidelines. We also present statistics of alleged and proven RCR violation cases and frequency of appeals to TENK on the decisions or procedures of the primary institutions. In addition, we analyze the available data on seven investigated cases in more detail. Positive aspects in the Finnish system are a fairly good infrastructure to investigate suspected RCR violations and a wide concept of RCR violations, which consists of fabrication, falsification, plagiarism, misappropriation, and other misbehaviors. However, the guidelines contain poorly elaborated definitions, do not treat the complainant and the suspect in an equal way, and need to be revised. Confusion about the concepts and criteria of the RCR violations seems to be common in primary institutions and among the complainants. Even if research institutions and universities have officially adhered to the national RCR guidelines, slipping from the guidelines occurs quite commonly. All these factors lead to frequent dissatisfaction with the decisions or procedures applied, high rate of appeals to TENK, and far from optimal functionality of the system.

## Background

Research misconduct in the form of fabrication and falsification is thought to be rare, though it is difficult to obtain a reliable estimate of the actual rate [1–4]. Plagiarism has been found to be common in master’s theses [5, 6], and questionable research practices are also common [1, 3, 7, 8]. There has been need to develop policies to promote research integrity by creating guidelines of responsible conduct of research (RCR), providing education on RCR, defining actions which represent research misconduct, and creating an organization to deal with these issues. Definitions of research misconduct (scientific misconduct, scientific dishonesty) and procedures to handle alleged violations of the RCR vary between different countries, and various practices may be in use even within the same country [9–13]. Several countries have established national agencies for the guidance of research integrity [9, 10, 12, 13], but still, a majority of the countries in the world lack guidelines and an organization to respond to research misconduct [14].

The Finnish Advisory Board on Research Integrity (in Finnish: Tutkimuseettinen neuvottelukunta, abbreviated TENK, portal www.tenk.fi [15]) began functioning in 1992 to promote research ethics and integrity through education and information, act as an expert body in making proposals and statements to authorities, give statements of alleged misconduct cases investigated primarily in universities and research institutes, and participate in international cooperation. TENK has a chairperson, vice chairperson, secretary general, and eight members representing different organizations and disciplines, jurisprudence included, and is appointed by the Ministry of Education and Culture. TENK released the first guidelines of research misconduct in 1994, and these have been later revised in 1998, 2002, and 2012. At present, all publicly funded universities, universities of applied sciences, and research institutes have formally committed to follow the guidelines. Alleged research misconduct cases are investigated at the local level of universities and research institutes, and a party not satisfied with the outcome or the process of the investigation may request TENK to give a statement of the case. TENK’s decisions and statements are recommendations, and the institutions are not legally obliged to follow them.

In this article, we present and evaluate the Finnish 2012 guidelines of RCR and misconduct in research. We also present data how commonly alleged violations of the RCR are reported and cases verified and evaluate how well the Finnish system works.

### Source of data and information

The www.tenk.fi portal presents the RCR guidelines and annual reports, and the 2012 guidelines are also translated in English [15]. As there are only minor differences in the definitions of various forms of misconduct in the 1998, 2002, and 2012 guidelines, we present only the 2012 guidelines here. Available numerical data and case summaries were retrieved from the annual reports; in some cases, more information was also obtained from the original documents and newspapers. All the information we have utilized is public on the basis of the Act on the Openness of Government Activities (621/1999).

### Responsible conduct of research and procedures for handling allegations of misconduct in Finland

#### Scope of application of the RCR guidelines

The purpose of the guidelines is to provide researchers with a model for the responsible conduct of research, define various violations against the RCR, and describe the procedure for handling alleged violations of the RCR. TENK does not deal with issues which belong to the judicial system or to the jurisdiction of other authorities and organizations.

Researchers should comply with the described principles of the RCR also when acting as teachers, instructors, scientific experts, and referees. These principles apply to publications; manuscripts sent to be published; abstracts; posters; applications for research positions and funding; referee statements; other written or spoken statements; evaluations of academic theses, textbooks, and other teaching materials; CVs; and publication lists, as well as to social interactions in both printed and electronic publication channels, including the social media.

#### Principles of responsible conduct of research

Responsible conduct of research is described on about one page in the Finnish guidelines, and this section mostly deals with issues at a general level. According to the characterization of the RCR, researchers are honest, meticulous, and accurate in their work; follow scientifically accepted principles in planning, performing, publishing, and evaluating an investigation and storing data; take other researchers’ achievements into account in an appropriate way; and acquire an ethical evaluation and a permit for studies requiring these. When starting a research project, an agreement defining each person’s position, responsibilities, and rights for research results and authorship is signed. Conflicts of interest are taken into consideration and reported in publications. Research organizations follow good personnel and financial administration practices.

#### Violations against the responsible conduct of research

Violations of the RCR consist of research misconduct and disregard for the RCR.

Research misconduct means scientific fraud and includes fabrication, falsification, plagiarism, and misappropriation (FFPM). FFPM refers to misleading the research community and often also to misleading the decision-makers. It includes presenting false data or results to the research community or spreading false data or results in a publication, presentation given in a scientific or scholarly meeting, manuscript to be published, teaching materials, or applications for funding. Research misconduct is divided into four subcategories.

Fabrication refers to reporting invented observations to the research community. The fabricated observations have not been made by using the methods as claimed in the research report. Fabrication also means presenting invented results in a research report.

Falsification refers to modifying and presenting original observations deliberately so that the results based on those observations are distorted. The falsification of results refers to the unfounded modification or selection of research results. Falsification also refers to the omission of results or information that are essential for the conclusions.

Plagiarism refers to representing another person’s material as one’s own without appropriate references. These include research plans, manuscripts, articles, other texts or parts of them, visual materials, or translations. Plagiarism includes direct copying as well as adapted copying.

Misappropriation refers to the unauthorized presentation of another person’s results, ideas, observations, or data as one’s own.

The terms “scientific community” and “decision-makers” are of importance in the definitions. A dishonest deed needs to be directed towards the scientific community or decision-makers in order to represent fraud.

Disregard for the RCR is defined to manifest itself as gross negligence and carelessness during the research process. Examples of the disregard for the RCR include belittling the role of other researchers in publications, such as neglecting to mention them and referring to earlier results inadequately or inappropriately, reporting research results and methods in a careless manner resulting in misleading claims, inadequate record-keeping and storage of results and research data, redundant publication, and misleading the research community in other ways. In this context, neglecting to mention other researchers means omitting their names from the Acknowledgements or not referring to them in the Methods or Discussion. When a researcher, who is entitled to authorship, is omitted from the list of authors, the case represents misappropriation and fraud.

#### Guidelines for handling alleged violations of the responsible conduct of research

Alleged violations of the RCR are investigated in the universities and research institutes where the alleged misconduct has taken place. If the suspected person is employed in more than one institution, they need to collaborate in the investigation. The investigation process involves three steps which are a written notification, preliminary inquiry, and investigation proper. During the preliminary inquiry, statements are obtained from the person suspected of misconduct, the initiator of the allegation, and, if necessary, from experts and other persons involved. The purpose of the preliminary inquiry is to initially determine the validity of the allegations. The rector or director must start the investigation proper if there is reason to suspect fraud or disregard for the RCR or the suspected person demands it.

The investigation proper is conducted by an investigation committee. The rector or director invites the members of the investigation committee and appoints one of them as the chairperson. The investigation committee must represent the necessary academic and legal expertise, and at least two of the members need to be external to the organization conducting the investigation. The investigation committee requests statements from the parties involved and, if required, holds oral hearings and gathers information from other sources. The investigation committee needs to release a final report on its work. On the basis of the final report, the rector or director decides whether an RCR violation has taken place and, if so, what corrective and punitive actions are taken. In a case of fraud, the ruling should be made public in an appropriate manner, and a copy of the decision sent to at least those publication channels in which fraudulent results or untruthful data were originally published. When necessary, TENK acts as an appeal body. The investigation procedure needs to follow the principles of the Administrative Procedure Act (434/2003).

### Statistics from TENK’s annual reports

During 15 years from 1998 to 2014 (years 2005 and 2009 are excluded, since some of the numeric data were missing from the annual reports), TENK received notifications of 142 cases of alleged RCR violations, and this corresponds to a mean of 9.5 cases per year. Fraud or disregard for the RCR was detected in 39 cases, which is 27.5 % of the notifications. The most common forms of the RCR violations were disregard for the RCR and plagiarism, representing 46.2 and 43.6 % of the violations, respectively, and in few cases, a person was found guilty of more than one type of misconduct. During the abovementioned 15 years, TENK gave 64 statements, which means that in 45.1 % of the cases TENK acted as an appeal body. Statements from TENK were requested mainly by the complainants, and the common reasons were as follows: preliminary inquiry was not done, the complainant was not adequately heard or TENK’s guidelines were not followed in some other way, the evidence was not taken into account accordingly, disagreement with the interpretation of the definitions of misconducts, and disputes on the rights for authorship and results. TENK declined to give a statement in some cases mainly due to the reasons that the issue did not belong to its jurisdiction or the primary institution had not yet finished the investigation. Since the annual reports are often deficient, no proper information could be retrieved regarding how often TENK disagreed with the decision or procedure of the primary institution.

### Case summaries with comments

We present examples of cases handled by primary institutions and TENK during 2009–2014 and representing decisions and views we disagree with or regard as insufficiently reasoned. The Finnish language does not differentiate between he and she, and in cases where the gender is not known, we use the words “he” and “his.” We are not involved as complainants or suspects in any of the cases.

Disclaimer: We have tried to present the views of all parties involved and have made our conclusions on the basis of the available data, yet our conclusions are opinions and not facts.

#### Case 1

A researcher (A), dissatisfied with the decision of the primary institution, requested TENK’s statement on the issue. A suspected his fellow researcher B of plagiarism and misappropriation, since B had utilized without permission the research plan presented confidentially to him. B had made a funding application to the Academy of Finland. In the application, he had cited without permission text from the other researchers’ previous application, mentioned that these other researchers are his collaborators in the forthcoming project, and kept these things secret during the 8-month period it took to handle the application and approve the funding. According to TENK, B was guilty of disregard for the RCR but not of misappropriation, because he had not presented the research plan as his own but mentioned the other researchers as collaborators.

We regard this decision from 2009 as wrong, and in our opinion, the deed clearly fulfills the criteria of fabrication. Presenting false information to a funding body was defined as fraud already in the 1998 guidelines. Besides, there is at least one earlier case and decision, in which presenting untruthful information about the collaborators to a funding body was regarded as fabrication.

#### Case 2

Senior researcher A had made a notification of alleged misappropriation after being excluded from the authorship of publications. According to the verdict of the university’s preliminary inquiry in 2012, B and C (leader and member of the joint project, respectively) were guilty of disregard for the RCR and A’s name should be added to the Acknowledgements of one article. Researcher A appealed to TENK, which ruled in 2013 that the preliminary inquiry did not deal with A’s right to authorship thoroughly enough, and the university needs to initiate the investigation proper. The rector appointed the investigation committee accordingly.

The public documents reveal how the investigation committee had difficulties in differentiating misappropriation from disregard for the RCR. The committee requested for TENK’s guidance in the form of previous misappropriation cases or appropriate literature, but TENK was not able to help. The committee members tried to clarify TENK’s definition of misappropriation with the help of definitions applied in foreign institutions and organizations and even utilized the Criminal Code of Finland to clarify the concepts of intent and gross negligence. The committee came to conclusions that misappropriation needs to be intentional, gross negligence is very near to intent, and a deed can be intentional also in a situation in which the offender regards a consequence as possible but does not try to prevent it or accepts the consequence.

According to the investigation committee’s final report issued in 2014, B was found guilty of disregard for the RCR, since she had not honored the legally binding authorship agreement with A, repeatedly refrained from informing A about the manuscripts, and belittled A’s contribution in the articles. Two members of the investigation committee did not find B guilty of misappropriation, because “she has not intentionally and thus illicitly presented A’s research findings or tried to use them in her own name.” One member of the committee regarded B’s actions as intentional, when she had repeatedly denied the existence of publication plans to A, even while manuscripts were being drafted, regarded her as guilty of misappropriation, and issued a divergent opinion on the matter. C was regarded as guilty of disregard for the RCR on the basis of neglect of duty and belittling A’s scientific work. B was obliged to add A to the authors of one article within 3 months; otherwise, she would be considered guilty of misappropriation. In addition, C was obliged to refer to A in the text of one article.

TENK’s decision on the necessity of the investigation proper was well reasoned, but unfortunately, TENK could not give further guidance to the investigation committee. In our opinion, when B untruthfully and repeatedly denied the publication plans and writing process to A, her intention was to exclude A from the authorship of the articles, and we agree with the member of the investigation team who regarded B’s conduct as misappropriation. Misappropriation was added to the Finnish RCR guidelines in 1998, but surprisingly few misappropriation cases have been detected over the years. There are actually several misappropriation cases in TENK’s annual reports, but they have been interpreted to represent disregard for the RCR.

#### Case 3

The unpublished results of researcher A’s master’s thesis had been utilized in scientific publications by other researchers without asking A to act as a co-author. A had sent notifications of alleged plagiarism to two institutions and later appealed to TENK. Both institutions had stated that no violation against the RCR had taken place, but A’s name should have been added to the publications as a co-author. According to TENK’s statement from 2011, the institutions had mismanaged the case in several ways: no proper preliminary inquiry led by the rector or director had been done, no investigation committee had been set up, A had not been heard during the investigation, no contra-accusation against A should have been presented in the decision, and TENK had not been notified of the investigation. TENK ruled that the investigation proper needs to be undertaken, since an RCR violation had not been ruled out and the concept of plagiarism applied by the persons investigating the notifications had not been consistent with TENK’s or international definitions. The case was then reinvestigated by the institutions, and in the final reports from 2012, the co-authors were found guilty of disregard for the RCR but not of plagiarism, when they had excluded A from the authorship in the publications. No corrective procedures are mentioned in the case summary, but presumably, the co-authors were obliged to add A’s name afterwards to the publications.

The issues raised in TENK’s statement concerning formal errors in the investigation appear to be correct, but it was less relevant to regard an incorrect concept of plagiarism as one reason to reinvestigate the case. According to the Finnish guidelines, when a researcher’s unpublished results are utilized without permission, there is reason to suspect misappropriation and not plagiarism, which is applicable to published results and text. It is probable that the supervisor of A’s master’s thesis was among the authors of the publications and at least he must have known that A was entitled to authorship. In our opinion, the co-authors who were aware of A’s right to authorship were guilty of misappropriation and not of disregard for the RCR.

#### Case 4

Several researchers had worked in a joint project with professor A. A had prepared a manuscript on the basis of their common results and made alterations in the final version of the manuscript without consulting the other team members. He had omitted researcher B’s name and shifted his own name as the first author. He had sent the manuscript to be published without asking the other researchers’ permission. A preliminary inquiry and investigation proper were done. According to A’s account, B’s contribution to the publication had been insignificant and of technical nature. The investigation team ruled in 2012 that A had belittled B’s work, B was entitled to authorship, and A was guilty of disregard for the RCR. It seems that none of the parties later requested TENK’s statement on the case.

We pondered two options and regarded the first one as more probable. Professor A acted in secrecy, because he did not want to face objection from the other team members, and he was aware of the consequences of his deeds, i.e., he acted intentionally. The second option is that A simply did not care about the rights and opinions of his fellow researchers, and this conduct represents recklessness. In our opinion, A acted either intentionally or recklessly, and therefore, his exclusion of B from the authorship represents misappropriation and not disregard for the RCR. There was also disregard for the RCR and towards all the other authors of the article when A listed himself as the first author and by doing so improved his position at the expense of the others, did not consult the fellow researchers about the order of the authors, and sent the final version of the manuscript to the journal without the co-authors’ consent.

#### Case 5

A lecturer (A) in a university of applied sciences had submitted an allegation of plagiarism against her two colleagues (B, C). After having received an unsatisfactory decision from the rector, A appealed to TENK. A had helped B and C to prepare their congress poster after finishing her own poster. A had sent B and C via email her final poster as a PDF file and the common poster template of their institution as a PPT file. A, B, and C attended the same international congress. After putting up the posters, it was noticed that the Background section of B’s and C’s poster contained exactly the same text as A’s poster, altogether 39 words. A requested B and C to cover the text they had copied from her poster, after which B covered six words of the text. The remaining text was quite general and would have been suitable also for the theme of B’s and C’s poster. A repeated the request to cover all the copied text, and B taped a white sheet of paper on it. The stepwise covering of the copied Background part in the poster is verified by the photos A took. B’s and C’s paper in the congress abstract book did not contain copied text.

The rector’s and TENK’s decisions were similar, and both were issued in 2012. No violation of the RCR had taken place. A had sent her own material to B and C, and these two had prepared their own poster on the same template. The same text in B’s and C’s poster had been left in its place from A’s poster by accident and not intentionally. As an additional proof for their decisions, the rector and TENK mentioned that the posters dealt with different topics and B and C had corrected their mistake without delay after having been notified about it.

We are surprised that the rector and TENK regarded this case as an honest error and did not see anything reprehensible in B’s and C’s conduct, when they failed to check and correct their poster before putting it up and needed two requests to fix the incident. In our opinion, B and C neglected the RCR criteria of meticulousness, accuracy, and respect for another researcher’s work, acted in a very careless way, and were therefore guilty of disregard for the RCR.

#### Case 6

Researcher A had sent a notification to a university and suspected B, the leader of the research team, of fabrication in a patient study. The report of the preliminary inquiry stated that B had included an inaccurate conflict of interest statement in a publication and reported in an inadequate way about laboratory test methods and the selection of patients into case study publications. It was also stated in the decision that a reader of the published data may not get a correct comprehension of the treatment. On the basis of the preliminary inquiry in 2012, B was found guilty of disregard for the RCR. Researcher A appealed to TENK. In the notification, A suspected B of falsification and complained that the preliminary inquiry team had refused to hear his witnesses, who could have been able to testify that B had denied them to speak about an expired patient’s case. According to A, the alleged misconduct should be investigated thoroughly in the investigation proper.

TENK found no rationale for the investigation proper for the following reasons. The primary inquiry had been comprehensive enough, and a more thorough investigation would unlikely add significant information to the case. There was no need to hear A’s witnesses. Even if B had demanded to keep the death of a patient secret, it cannot be concluded that B would have intentionally tried to mislead the scientific community. In situations where a publication has been stated to deal with only one patient case, it cannot be stipulated that also other cases are reported. Omitting other possible cases from the publication does not represent fabrication of the findings, and a researcher has the freedom to make this kind of a choice.

According to TENK, a researcher was free to choose and publish only one case in a report even if more than one case was investigated. We highly disagree with this decision from 2012 and see a danger of falsification lurking in such practice. Patient(s) can be excluded from a study and publication but only if the researcher follows scientifically based exclusion criteria. Even the preliminary inquiry raised a concern that B’s manner of presenting data may mislead the readers. TENK should have recommended the investigation proper in this patient study.

#### Case 7

The case has been reviewed in Retraction Watch [16]. A leader (A) and a member (B) of a large research group filed a complaint to TENK criticizing the way the research institute had handled their case of alleged violation of RCR. A and B complained that they had not been informed why an investigation team was formed to scrutinize a specific publication of their research group and who was the initiator of the allegation. After the investigation proper, the director of the institution had made a decision in 2014 stating that no violation of RCR had occurred, even though the publication examined contained exaggerated conclusions concerning the metabolomics part.

TENK supported A’s and B’s view that they had not been accordingly informed about the investigation and recommended the research institute to check their procedures for the future. TENK also stated that exaggeration of the conclusions in the publication appeared to belong to the field of scientific dispute and not to represent an RCR violation.

The research institute handled the case originally poorly, it did not follow the guidelines to investigate suspected violations of the RCR, and neither did it specify what “exaggerated conclusions” really meant. TENK did not recommend a new investigation, even if A and B had not been adequately heard during the investigation and no legal and medical expertise had been included in the investigation team. According to the principles of the RCR, researchers should be honest, meticulous, and accurate. If a researcher presents exaggerated conclusions, there is a danger of misleading the scientific community. TENK should have paid more attention to the national guidelines and recommended a new, thorough investigation due to formal errors in the previous investigation and to find out with the help of scientific experts what “exaggerated conclusions” really include. The case has aroused a lot of interest in Finland, and the leadership of the institute has announced to reinvestigate the case.

## Discussion

The numeric data from TENK’s annual reports indicate that universities and research institutions have handled on the average 9.5 cases of alleged RCR violations per year in 1998–2014 (years 2005 and 2009 excluded due to insufficient data), and the allegation has proven correct in 39 (27.5 %) cases, on the average 2.6 cases annually. For comparison, the Austrian Commission for Research Integrity has discovered 11 cases of research misconduct or violations of good scientific practice in 2009–2013, on the average 2.1 cases annually [17]. In Denmark, 7 new cases of scientific dishonesty were found in 2011–2015, on the average 1.4 cases annually [18]. It is noteworthy that the Danish definition of scientific dishonesty is a bit narrower than the Finnish definition of RCR violations. These small numbers of proven misconduct cases represent underestimation, because a majority of the cases probably go unnoticed, and of the noticed cases, not all are notified [2, 4].

The results also show that in 45.1 % of the cases one party, usually the initiator of the allegation, has obtained TENK’s statement on the issue. On the basis of this high rate of appeals, we conclude that dissatisfaction with the decision or the procedure of the primary institutions is common. We cannot provide any numeric data on the complainants’ satisfaction with TENK’s decisions, but making an appeal to TENK probably leads to a satisfactory outcome in the majority of the cases. Still, dissatisfaction with TENK’s decisions is not rare, and this may be due to several factors. There is disagreement between TENK and the complainants on how to differentiate, e.g., misappropriation and plagiarism from disregard for the RCR and the latter from minor errors. There is also inconsistency in TENK’s decisions. When a primary institution has not followed the official guidelines, TENK often recommends a new investigation but not always, e.g., reinvestigating the Case 7 was not proposed. TENK makes occasionally decisions which are in contradiction with the guidelines, like in Cases 1 and 5. TENK should not make presumptions which require specific scientific expertise as it has done, e.g., in Case 6.

Reluctance to start an investigation or do it properly is not rare in the primary institutions. Obviously, there is fear of negative publicity and loss of research funds, and the investigation process also demands time and money. The rector may perform the preliminary inquiry even alone and may interpret incorrectly the concepts of RCR violations or lack the necessary scientific expertise for the task. Confusion about the concepts and criteria of the RCR violations seems to be common in primary institutions and among the complainants, and this is illustrated, e.g., in Cases 1, 2, and 3. There are several reasons for this, such as allegations of RCR violations are not everyday matters in universities and research institutes, TENK’s guidelines contain poorly formulated definitions, and TENK’s case summaries from the annual reports are often too brief and superficial to provide really informative educational material.

It is a positive thing that the Finnish definitions cover many other misdemeanors in addition to FFPM, but there is scope for improvement. Good guidelines are precise, comprehensive, and consistent and take into account the ethical values and legislation of the society. The Finnish guidelines for research integrity and misconduct do not fulfill all these criteria in a satisfactory way and lack an analytical approach to the topic. The definitions of the RCR violations consist of the research misconduct proper or fraud (FFPM) and disregard for the RCR, which is regarded as a milder form of misconduct than fraud. Fraud means cheating the research community or decision-makers, while deceiving fellow researchers or acting in a grossly careless or negligent way represents disregard for the RCR. The classification does not represent well the seriousness of the deeds in a moral sense, since dishonest, unethical, and even illegal acts are included in the category of the disregard for the RCR. We do not see any rational basis why dishonest deeds directed towards colleagues and their research, such as fabricating results and distributing them to colleagues, even sabotaging their work, represent a milder misconduct than cheating the scientific community and decision-makers. Neither do we agree that breaking the laws and regulations by mistreating the research subjects or endangering the environment are less serious misconducts than FFPM. The Finnish RCR guidelines are also flawed in the unequal way how the complainant and the suspect are treated. The investigation proper has to be started if the suspect requests it, while the complainant does not have the same right. This specific issue in the guidelines is against the equality principle guaranteed to us in the constitution.

When interpreting the Finnish RCR guidelines, it is often presumed that scientific fraud needs to be intentional, and this is illustrated well in Case 2. Actually, the guidelines do not postulate intention, they state that RCR violations, viz. fraud and disregard for the RCR, take place either intentionally or out of carelessness. On the basis of the existing definitions and common sense, one cannot conclude that research fraud is always intentional and disregard for the RCR always shows gross negligence or carelessness but no clear intentionality. Fraud is often intentional but not always, and disregard for the RCR may also be intentional. For example, plagiarism, if not extensive, may happen out of carelessness and disregard for the RCR, like belittling another researcher’s work by not mentioning his or her achievements in an article, may happen intentionally or knowingly. When interpreting the Finnish research fraud definitions, we could use the widely adopted prerequisites for intent in the research misconduct definitions of the European Science Foundation and All European Academies [19] and the US Office of Research Integrity [20]. Both guidelines state that research misconduct consists of actions which are done intentionally, or knowingly, or recklessly. Intentionality means acting on purpose to cause a certain consequence; acting knowingly means that a person considers a consequence of his or her action certain or quite probable, and a person showing reckless behavior does not think and care about the consequences of his or her actions.

Poorly formulated definitions of the RCR violations need to be revised in the Finnish guidelines. It is not just observations and results which can be fabricated, but researchers can also lie when presenting their materials, study populations, methods, scientific merits, collaborators, acquisition of study permits, and other issues. Likewise, falsification does not occur only by the unfounded selection of observations or results, but the message of a scientific study can be distorted by a biased selection of research materials, study populations, and methods, including statistical methods. In the introductory part, disregard for the RCR is said to manifest itself as gross negligence and carelessness during the research process. Here one example of disregard for the RCR has been used to characterize the whole category, even if also deeds showing intentionality or conducted as teachers or scientific experts outside the actual research field may belong to this category. We do not support dividing research misconduct into the misconduct proper and disregard for the RCR, since we regard this division as artificial and confusing. We prefer a simple classification in which fraud and other violations of the RCR are listed under the same title of research misconduct. The severity of the deeds vary, but this can be taken into account in the sanctions. There is a decent definition of research misconduct presented by the European Science Foundation and All European Academies [19]. It includes FFP and other forms of misconduct which fail to meet clear ethical and legal requirements, and it could be further improved and perhaps modified for national purposes.

The RCR guidelines contain an important principle which states, “if a violation of the RCR has occurred, the sanctions for that violation must be in just proportion to the severity of the violation.” Disregard for the RCR contains various misconducts, some of which may be dishonest, unethical, or illegal, yet the RCR guidelines do not take this into account. An obligation or strong recommendation to publish the misconduct cases applies only to FFPM. Our view is that the proven cases of disregard for the RCR should be published as well, at least on the official forums of the universities, and possible funding bodies should also be informed. At present, the punitive actions from disregard for the RCR tend to be mild, and in some cases, there has been no sanction at all. Research fraud may lead to rejecting a dissertation, revoking an academic degree, loss of funding, a severe warning, or a temporary dismissal of a student from a university, but cases in which persons have been dismissed from their posts due to research fraud are rare.

## Conclusion

We have a national agency functioning in the wide field of research integrity and ethics, and the situation in the universities and research institutes would probably be much worse in the absence of TENK’s overseeing. However, the Finnish system to promote research integrity and handle alleged violations of the RCR does not function in the best possible way due to many reasons. In spite of the fact that the institutions have officially adhered to the national guidelines of research integrity, some lack of commitment is not rare. The institutions do not always follow the national guidelines, and the investigations are at times done in a sloppy way. There is also scope for improvement in the guidance provided by TENK. The official guidelines need to be revised since they do not reflect well the ethical values of the society, treat the complainant and suspect in an unequal way, and contain poorly formulated definitions. Case summaries on TENK’s website need to be more informative and detailed for educational purposes. A course of research integrity and misconduct should become obligatory for all students in universities and universities of applied sciences. We also need local research integrity advisors who would be useful in settling disputes, providing information, and supporting whistle-blowers and those suspected of research misconduct. Universities and research institutes should publish all proven cases of misconduct and other misdemeanors in research, since the decisions are public documents by law and negative publicity is often the only sanction the culprit gets.

Note added: After sending this manuscript to *Research Integrity and Peer Review*, TENK announced a report which had been requested by the Ministry of Education and Culture. The report contains proposals to facilitate notifying on alleged research misconduct, protect whistle-blowers, and promote education, information, and counseling on research integrity and misconduct issues, like appointing research integrity advisers in organizations and launching a Research Ethics Library website. All these proposals are important and worthwhile, but still, many issues in the present RCR guidelines warrant revision.

## Response from the Finnish Advisory Board on Research Integrity, TENK

Sanna Kaisa Spoof (Secretary General), Krista Varantola (Chair), and Pekka Louhiala (Vice Chair) of the Finnish Advisory Board on Research Integrity, TENK

### General clarification of the role of the Finnish Advisory Board on Research Integrity, TENK

The philosophy behind the Finnish system of handling alleged misconduct is based on self-regulation and the principle that science corrects itself. This philosophy is put into practice by means of the guidelines: *Responsible conduct of research and procedures for handling allegations of misconduct in Finland. Guidelines of the Finnish Advisory Board on Research Integrity 2012.* These guidelines were devised in cooperation with the Finnish science community (science is used in its widest sense and covers all disciplines and thus also social sciences and humanities). All higher education institutes and research institutes in Finland are committed to following the guidelines. In other words, by the institutional signatures, they have given a collective oath and are committed to adhering to the guidelines and the procedures described in them when conducting investigations of alleged misconduct cases. They are also required to inform the Finnish Advisory Board on Research Integrity (TENK) of all ongoing investigations. The signed institutional commitment is a unique feature of the Finnish system. Overall, the institutions take this commitment very seriously and follow the instructions meticulously. The guidelines are available in English at www.tenk.fi

Normally, the guidelines are applied to post-MA-level research (doctoral studies, theses, and post-doctoral research). MA-level theses are sometimes included in the investigation, if misconduct is suspected “post-factum.” Dealing with misconduct allegations at undergraduate (BA/MA) level studies belongs to the universities and is not brought to the attention of TENK.

TENK can be described as an appeals court without being a court. The parties not satisfied with the procedure or the result of the university investigation can request a statement from TENK (the ten-member Board). In its statements, TENK takes a stand only in those issues of the investigation process that are specified in the request. TENK does not comment on matters of opinion such as disputes between different schools of thought or on issues of professional ethics (medical ethics, biotechnology, etc.).

TENK may ask an institution to conduct a full investigation after a preliminary investigation, if it thinks that a full investigation with an external investigation committee will bring more light to RI issues and be in the interest of the parties involved in the investigation. This does, however, not mean that TENK has at this stage sided with any party involved.

The investigation of any allegation of misconduct is in the hands of the institution/s involved (self-regulation philosophy), but the guidelines give very exact instructions about the procedural rules to be followed in the preliminary investigation and the investigation proper. Contrary to the claims made by Liisa Räsänen and Erja Moore, TENK does not explicitly advise the investigating institution on how to run the investigation and how to decide on the type of misconduct that is being investigated. This type of advice would result in a conflict of interest situation if there is a follow-up request to TENK.

The members of TENK always declare all potential conflicts of interest that may arise in any case that TENK has been asked to comment on.

### The purpose of the guidelines

The TENK guidelines are *guidelines* that help in the investigation of alleged breaches against RI. Guidelines need to be interpreted and applied in individual contexts. To help the investigation committees in their work, the guidelines include examples of irresponsible practices.

The guidelines are in a sense the “legal” RI framework which experts use to reach their judgments in RI cases, which are usually far from straightforward and need to be scrutinized from a variety of angles.

The guidelines are valid for all disciplines and are applied for all types of allegations of irresponsible practices in research.

The authors claim that the guidelines do not make a clear enough distinction between misappropriation and disregard for responsible conduct of research. It should be kept in mind that in many international guidelines misappropriation is treated as a subcategory of plagiarism. In Finland, misappropriation has been kept as a fourth type of misconduct in addition to the traditional FFP division.

Disregard for responsible conduct of research is a category used in Finland to deal with gross negligence and carelessness in the conduct of research. It includes among things authorship issues and reporting research results in a careless manner resulting in misleading claims.

Due to the differences in the categorization of breaches against RI in different countries, comparisons between countries and the available statistical information are difficult and need to be treated with major reservations.

In its annual reports, TENK gives short summaries of the cases it has been dealing with during the year in question. They are anonymized and do not go into details. The cases are never identical, and the categorization of the breach depends on a number of factors.

The full discussion and the reasoning behind the conclusions are available in the final statements. The final statements are public and can be studied at request.

TENK does not issue sanctions. Sanctions are in the hands of the investigating institution.

### The argumentation by Räsänen and Moore

In most cases, Räsänen and Moore base their claims on the anonymized summaries and they have not read the full statements. In some unspecified cases, they have had at their disposal additional information which is not part of the official documentation. This information is not included in the references. Furthermore, they do not clearly specify what information they have used in specific cases. Neither do they in any way explain why their judgment is more competent than that of the investigation committees and the TENK Board.

Räsänen and Moore compare the number of cases during a specific period. However, they do not take into account that the guidelines were updated during that period. The latest guidelines also take into account the so-called gray area practice in research, such as “upgraded” CVs and lists of publications used for self-promotional purposes. This has increased the number of reported allegations.

The main reason behind the rise in the number of allegations in the past few years is, however, mainly due to authorship disputes.

The claim that 45.1 % of the researchers involved in the investigation processes are unhappy with the guidelines and the processes is not based on facts. This figure is based on the number of allegations reported to TENK and the number of statements requested from TENK.

It must be remembered that the number of reported cases is not the same as the number of processes. One case can generate several allegations from the parties involved in the case. In addition, if project teams are involved in the allegation, they may represent several research organizations which will thus conduct the investigations concerning their own staff.

Furthermore, it must be taken into account that it is not necessarily the guidelines that are at fault if a party in the investigation is unhappy with the result. We can predict with confidence that there is in most cases one dissatisfied party after the investigation has been completed.

No guidelines and processes are perfect. At TENK, we are very happy that the Finnish process of investigating irresponsible research practices receives critical attention. We are very well aware that a great deal still needs to be done to promote good research practices. However, a poorly argued analysis is not helpful in this endeavor.

## Abbreviations

FFPM: Fabrication, falsification, plagiarism, and misappropriation
RCR: Responsible conduct of research
TENK: Finnish Advisory Board on Research Integrity, Tutkimuseettinen neuvottelukunta in Finnish
